# Supplementary material for: Bright light therapy and early morning attention, mathematical performance, electroencephalography and brain connectivity in adolescents with morning sleepiness
Source: PLoS One. 2023 Aug 22;18(8):e0273269. doi: 10.1371/journal.pone.0273269 (PMC10443881; doi:10.1371/journal.pone.0273269)
Supplement: S1 File — Originally submitted protocol for funding of this study. (PDF) [file pone.0273269.s009.pdf]

Martin H. Teicher, M.D., Ph.D
Director, Developmental Biopsychiatry Research Program
Chief, Laboratory of Developmental Psychopharmacology
McLean Hospital
115 Mill Street
Belmont, MA 02478-9106

Harvard Medical School
Department of Psychiatry
Associate Professor
Phone: 617-855-2970
Fax: 617-855-3712
Email:
martin_teicher@hms.harvard.edu

Revised Protocol

Effects of Litebook EDGE™ Phototherapy on Academic Performance and Functional Brain Activity in Non-Depressed Adolescents

November 3, 2016

Adolescence is characterized by a number of important biological changes, including myelination of corticolimbic pathways, pruning of synaptic connections, and alterations in brain metabolism, sleep quality and circadian phase[2,9,13,16,30,33,38,57]. As children pass through puberty, their circadian acrophase shifts, and they experience a strong urge to stay up and awaken late[21]. Hence, a large percentage of normal adolescents arrive at school each day with an insufficient amount of sleep[48,53], which can take a substantial toll on their academic performance[14,22,32,48].

A growing number of human studies clearly show that sleep promotes learning and memory[11,12,24,62]. Conversely, sleep deprivation has a negative impact on cognitive and behavioral functions[7,28,34]. Functional MR imaging (fMRI) studies comparing well-rested and sleep-deprived adults show that alterations in cognitive ability correlate with alterations in functional brain activity[19,63]. For example, fMRI studies reveal deficient prefrontal cortical activation in sleep-deprived individuals performing a serial subtraction task, which requires working memory and attention[27]. In contrast, fMRI studies show a decreased degree of activation in the medial temporal lobe (MTL)[26] and in bilateral posterior hippocampal regions[64] during tasks that require encoding of new learning into long-term memory. Sleep deprivation also leads to a compensatory overactivation in prefrontal and parietal attention networks during learning of new material, which occurs as a partially effective attempt to compensate for diminished hippocampal processing through increased attention and effort[18,26]. Another form of compensation during learning tasks in sleep deprived individuals is the exacerbated deactivation of the resting state default mode network[18,20,36], which is normally involved in self-referential thinking. Similar findings of prefrontal overactivation and exaggerated deactivation have been reported in a pilot fMRI study of sleep-deprived adolescents[8]. Together these findings confirm that sleep loss is associated with alterations in working memory, attention, and capacity for memory formation[62], and that these impairments can be visualized by alteration in fMRI response in selective brain regions.

Relatively few studies have examined effects of sleep deprivation on cognitive performance in adolescents (for review see[23,41,44]). In these studies total sleep deprivation was associated with impaired memory performance and diminished computational speed[46,56], while, partial sleep deprivation was associated with deficits in reasoning[45] and verbal creativity[47]. For example, male adolescents sleeping more than 8 hours per day had significantly higher reasoning ability than their peers who slept for less than 8 hours per day[45]. Some studies[15,47] reported that simpler cognitive processes such as working memory and computational speed may not be significantly affected by a single night of sleep limited to 4 to 5 hours. However, even mild sleep restriction of an hour or more,

when persistent across days, can lead to memory problems as severe as seen following total sleep deprivation[7].

The sensitivity of the adolescent brain to subtle sleep impairments was highlighted in a study where 12-14 year olds were allowed to play stimulating computer games or watch TV right before bedtime. This experience prolonged sleep latency, increased stage 2 sleep and reduced slow wave sleep. This modest degree of sleep restriction significantly impaired verbal memory consolidation[29]. Suboptimal sleep duration in adolescents was also associated with poor performance on a serial digit-learning test[51] during morning testing sessions, but not in afternoon sessions. Between 58-68% of high school students surveyed in Ontario report that they feel "really sleepy" between 8 and 10 A.M.[32]. Thus, achievement in early morning classes may suffer the most in sleep-deprived adolescents.

Fortunately, sleep only needs to be extended by a modest amount to enhance cognition in children. Sadeh et al[52] showed that performance on memory, attention and vigilance tasks in children improved significantly after 1 hour of sleep extension on three consecutive nights. Gais et al[31] and Backhaus et al[4] have also shown the beneficial effects of sleep on memory consolidation in children and adolescents.

The extent of the sleep deficit in children and adolescents can be quantified using actigraphs, which are wrist-watch sized devices that count and store the number of body movements (accelerations) across time in memory. The activity time series can then be downloaded and analyzed by algorithms to provide estimates of sleep latency, total sleep duration and sleep efficiency[1,50]. Steenari et al[55] reported that longer sleep latency and lower sleep efficiency were associated with decreased auditory and visual memory performance. Shorter sleep duration was associated with reduced working memory performance.

In summary, a modest reduction in sleep duration over the course of a school week exerts a cumulative toll, with neurocognitive effects similar to that produced by total sleep deprivation. Sleep deprived adolescents appear to be unaware of the consequences of sleep loss on memory and academic performance[46]. Sleep optimizes the consolidation of newly acquired information in memory. Hippocampal-dependent (declarative) memories benefit primarily from slow-wave sleep (SWS), whereas memories not depending on the hippocampus (procedural or emotional) show greater gains over periods containing high amounts of rapid eye movement sleep[11]. Sufficient sleep is also critical for the encoding of new learning prior to consolidation. One night of sleep deprivation markedly impairs hippocampal function, imposing a deficit in the ability to commit new experiences to memory[62]. Overall, there is compelling scientific evidence that schoolchildren, particularly adolescents, are chronically sleep deprived, that the degree of sleep restriction they experience exerts demonstrable effects on memory encoding, consolidation and processing speed, and that even a modest increase in sleep will result in measurable improvements in cognitive function. We also know that the primary reason that adolescents are sleep deprived is due to a naturally occurring phase delay in their biological clock, resulting in a propensity to stay up until late in the evening which is incompatible with the early rise times schools typically require. Finally, we know that light treatments at the appropriate time can phase advance the biological clock, potentially reversing this problem.

The hypothesis that we propose to test is that consistent morning use of the Litebook Edge™ phototherapy device, coupled with one-hour pre-bedtime use of blue-wave light blocking glasses while watching video screens, by normal adolescents will shift the circadian phase of their normal (nycthemeral) rest-activity rhythm. This in turn will enable them to fall asleep earlier and to receive an increased amount of sleep during the school week. They will consequently awaken more readily, feel

more awake during early classes, and will perform better on tests of academic performance, attention and working memory. Light therapy will enhance intrinsic default mode network (DMN) connectivity and strength of the reciprocal connections between the DMN and attention networks[De Havas, 2012 #36238]. Light therapy will also enhance resting state functional connectivity (rsFC) between thalamus and cortical regions[Killgore, 2015 #36230] and result in a measureable increase in size of the dentate gyrus[Kwon, 2013 #36247]. Degree of improvement in cognition, attention and functional and structural MRI measures will be directly related to average time spent each day activating (and hopefully using) the device, which will be independent variable in the statistical analyses.

Subjects recruited for study will be normal adolescents of either sex, age 14-18. They must be unmedicated (other than acne medications, albuterol inhaler, birth control pills, or other medications that are non-sedating and have no known cognitive or chronobiological effects as approved individually by the PI) and have no significant psychiatric symptoms on rating scales (BASC-2/BESS[65]). Subjects will also need to have an IQ > 80 and be enrolled in school (not home schooled). Individuals who undergo athletic training (e.g., football or ice skating practice) prior to the start of the normal school day, or other activities that may markedly influence their degree of morning alertness, will be excluded. To be enrolled subjects must indicate that their academic potential exceeds their current academic performance, and that they would be willing to consistently utilize a device that may enable them to perform better at school. We will also *exclude individuals with macular degeneration, diabetic retinopathy, retinitis pigmentosa, who have undergone laser corrective eye surgery in the past thirty days, who have been instructed by an eye doctor to routinely wear sunglasses when outside, or are taking photosensitizing medications (though most photosensitize to UVA and UVB, not blue light).*

All subjects will be provided with Litebook Edge™ and blue-light blocking glasses in order to undergo three weeks of early AM phototherapy (30 minutes) prior to school departure. The litebooks will record extent of activation throughout this period and we will determine if degree of ‘use’ has a significant influence on outcome measures. Baseline assessment will include 7 days of activity monitoring and sleep-wake diary recordings to determine acrophase and total sleep time during the school week and weekends. Testing will take place at the end of the school week (Thursday, Friday or Saturday AM) to assess the cumulative effects of sleep restriction. They will be tested on the Quotient ADHD System[58-60] to assess attention. The Permanent Product Measure of Performance (PERMP) test will be used as a treatment-responsive measure of academic performance and processing speed in mathematics[10], along with a sleep-deprivation sensitive Serial Addition/Subtraction task[61]. Degree of sleepiness will be assessed using the Epworth Sleepiness Scale[Johns, 1991 #36267] and IQ will be assessed using KBIT-2. At the end of this baseline week a subset of subjects (~ 50%) will be scanned (3T Siemens Prisma Scanner or 3T Siemens TIM Trio) in the early morning (8 – 10 AM) with sequences for volumetric T1-weighted morphometry (for assessing dentate gyrus volume), and BOLD functional connectivity analysis during resting state[17,35,42] and during performance of a Go/No-Go Attention Task[59]. We predict that subjects who are drowsy in the morning will have decreased resting state functional connectivity between their thalamus and portions of cortex, particularly motor, sensory-motor and tempero-occipital cortex as well as ventromedial prefrontal cortex[Killgore, 2015 #36230]. Further, we predict that they will have decreased intrinsic connectivity within their default mode network (DMN) and between their DMN and anticorrelated task-oriented network both at rest and during task performance[De Havas, 2012 #36238], as well as reduced dorsolateral prefrontal [Hager, 1998 #36263], and anterior cingulate cortex[Tana, 2010 #36266] activation during sustained attention blocks and in prefrontal, parietal, occipital and thalamic regions during attention lapses within blocks[Chee, 2008 #7865].

Traditionally, electroencephalography has served as the gold-standard for assessing alertness, drowsiness and sleep, and EEG spectral parameters can provide a minute-to-minute index of

vigilance and alertness[39,43]. EEG beta activity is a frequently used measure of alertness and was found to be greater under bright light than dim light conditions[5]. More recent composite indices of alertness include the maximum fractal length (MFL) of the EEG waveform[3] and the global field power (GFP) of upper alpha band (10-12 Hz) oscillations[25,49]. Hence, we will also include quantitative EEG assessments to complement the MRI protocols.

During the 3rd week of bright white light treatment they will undergo a week of activity monitoring followed by reassessment at the end of the school week using Epworth Sleepiness Scale, Quotient, PERMP, and Serial Addition/Subtraction. The subset who had baseline MRI scans will receive between 8-10 AM a post-treatment MRI scan for using the same sequences and task. All females will be tested prior to MRI for pregnancy. Only the child will be told the results of their pregnancy test. All subjects will also receive a repeat EEG assessment outside the scanner.

Overall, we are proposing to perform a large number of assessments as no one has previously studied the effectiveness of phototherapy in enhancing the cognitive performance of typically developing adolescents. Hence, the goal of this study is to not only test whether light treatment enhances brain activity and cognitive function, but to help determine which components and measures are most significantly improved.

Statistically, we will use linear mixed effect models[54] to ascertain if we can reject the null hypothesis that within subject differences from baseline to post-test in cognitive measures, sleep-wake parameters and neurobiological processes were unrelated to duration of use of the Litebook Edge™ during the 4 -week assessment period. Instead, we predict that there will be a significant linear relationship between average duration of early AM bright light therapy use and: total sleep time, measures of sustained attention on Quotient; performance on PERMP and Serial Addition/Subtraction; size of dentate gyrus; thalamocortical functional connectivity, default mode connectivity; prefrontal cortical activation during attention testing and EEG measures of alertness.

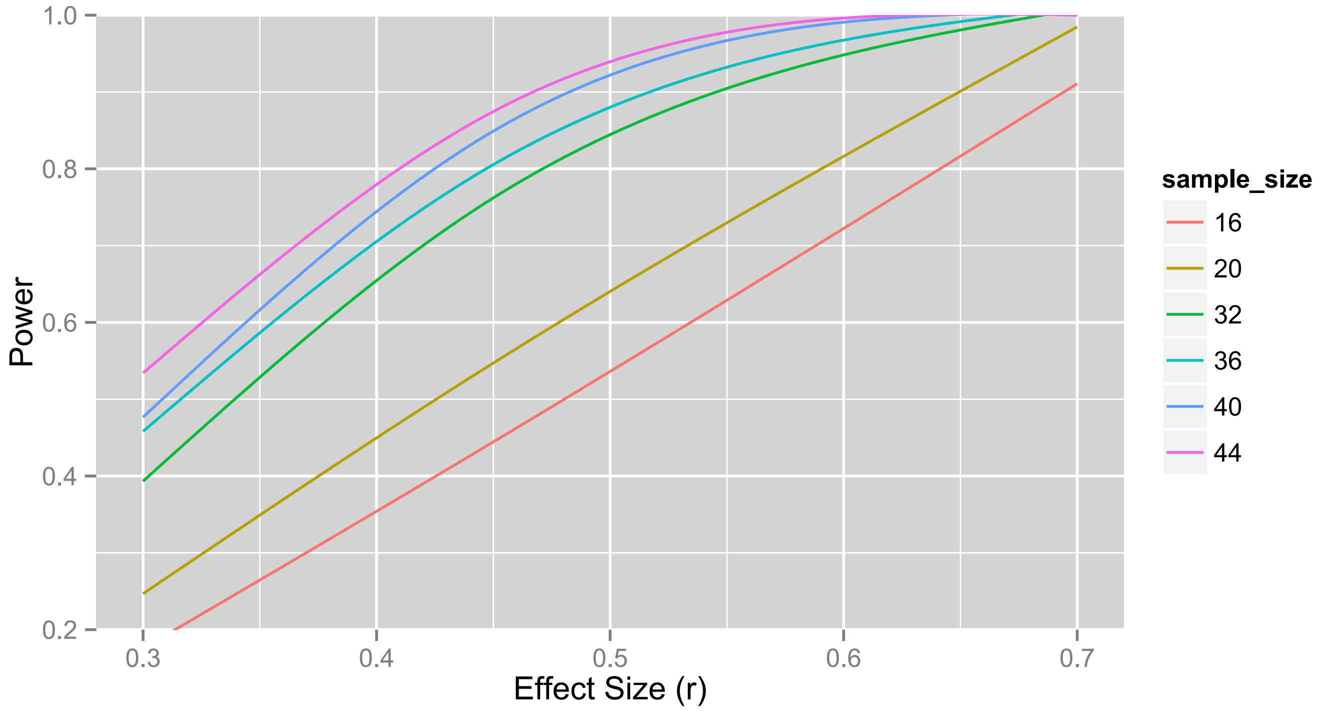

Figure 1 presents results of a Monte-Carlo simulation designed to assess power to detect a significant relationship between duration of Litebook Edge™ use and change in pre-to-post measures of attention, cognitive performance, sleep, MRI and EEG. The key determinant is the strength of the

relationship between degree of use and change score. A medium effect (r=0.3) implies that duration of LiteBook Edge™ use would account for 9% of the variance in change scores. A strong effect (r=0.5) would account for 25% of the variance. Randazzo et al.,[47] found that even a single night of restricted sleep (5 hours) resulted in moderate or large statistical effects on reaction time, memory, and creativity in children. Cumulative effects of mild sleep deprivation in school children appears to exert comparable effects on neurobehavioral function[22,51].The greatest impact appears to be on measures of sustained attention, and are associated with large effect sizes[Lim, 2010 #36285]. For neuroimaging measures, which show less session-to-session variability than behavioral measures, r-values of 0.6 (36% of variance) and 0.7 (49% of variance) are quite possible. Killgore et al[Killgore, 2015 #36230] reported correlations ranging from 0.519 – 0.614 between degree of drowsiness on the Epworth Sleepiness Scale in healthy non-sleep deprived individuals and measures of thalamocortical functional connectivity.

With n=36 subjects we would have an 80% chance of detecting a moderate-to-large correlation between duration of use and improvement in performance (r=0.45). With n=24 neuroimaging subjects we would have an 80% chance of detecting a large (r=0.6) association between duration of use and change in MRI parameters.

As this is a proof of concept study, we will consider all of the following to be planned comparisons and will not correct for multiple comparisons within the submitted report.

- Actigraph measures (3 comparisons)
    - Total sleep duration
    - Circadian acrophase
    - Mean activity first 2 hours after awakening
- Quotient measures (5 comparisons)
    - Errors of Omission
    - Errors of Commission
    - Percent time spent fully attentive
    - Variability In response latency
    - Response time (1/RT)[Basner, 2011 #36282]
- Performance measures (4 comparisons)
    - PERMP – accuracy
    - PERMP – speed
    - Serial Addition/Subtraction task – accuracy
    - Serial Addition/Subtraction task – speed
- MRI measures (44 total comparisons)
    - Volume of right and left dentate gyrus (2 comparisons)
    - Resting state functional connectivity between right/left thalamus and right/left motor, sensory-motor, tempero-occipital cortex and ventromedial prefrontal cortex (16 comparisons)
    - Default mode network (DMN) size, intensity at rest and during attention test (4 comparisons)
    - Degree of connectivity between DMN and right/left attention networks, central executive network at rest and during attention test (6 comparisons)
    - Right and left dorsolateral prefrontal cortex, anterior cingulate, occipital and thalamic activation during attention testing (block and event models) (16 comparisons)
- EEG (4 comparisons)
    - Theta activity
    - Beta activity
    - Maximum fractal length (MFL) of the EEG waveform
    - Gobal field power (GFP) of upper alpha band (10-12 Hz) oscillations

Note: with 3 planned comparisons probability of detecting one or more $p<0.05$ significant differences is 0.992 when the probability of detecting any difference is 0.8, and is 0.875 when the probability of detecting any difference is 0.5. Hence, this approach should yield multiple significant findings even with a relatively small sample size. Because of the possibility of false positives it would be desirable at some future time to replicate these findings in an independent sample.

This approach of using duration of device activation as the independent variable provides several advantages over comparing bright white light to placebo red light. First, effect size measures previously calculated assumed that subjects in both groups would actually use the devices. There will likely be significant variability between subjects in degree of use and if only a fraction of subjects assigned the bright white device used it consistently then the overall impact would be weaker and possibly missed in a two group analysis. Using duration of device operation will enable use to compare subjects who used it to a considerable degree versus subjects who hardly use it at all, and would likely provide a good estimate of how much benefit accrues from different degrees of use.

This is particularly important for the neuroimaging component. If we compared active versus placebo devices then only half of the neuroimaged subjects would receive the active device, which may leave us comparing pre versus post effects in only 8-10 subjects. In this revised design all of the neuroimaged sample (n = 24) would receive the bright white Litebook and blue-blocking glasses making the pre-post comparisons stronger, especially when adjusted for duration of device activation.

Second, using duration of device activation as the independent variable will markedly facilitate recruitment. If we used a placebo device we would need to indicate in the informed consent that subjects may receive a placebo device, though we do not reveal what the placebo is. Instead, we can now indicate in the informed consent that all subjects will receive a device that we believe is biologically active and that no placebos will be used.

This also makes the protocol simpler as raters do not need to be kept blind to device type. All we need to do is make sure that raters remain unaware of duration of device activation.

I’m also confident that the revised statistical approach of presenting these as planned comparisons will identify a substantial number of significant cognitive and neurobiological benefits.

References

1. Acebo, C., Sadeh, A., Seifer, R., Tzischinsky, O., Wolfson, A.R., Hafer, A., and Carskadon, M.A. (1999) Estimating sleep patterns with activity monitoring in children and adolescents: how many nights are necessary for reliable measures? Sleep 22, 95-103
2. Andersen, S.L., Thompson, A.T., Rutstein, M., Hostetter, J.C., and Teicher, M.H. (2000) Dopamine receptor pruning in prefrontal cortex during the periadolescent period in rats. Synapse 37, 167-169.
3. Arjunan, S.P., Kumar, D.K., and Jung, T.P. (2010) Estimation of alertness levels with changes in decibel scale wavelength of EEG during dual-task simulation of auditory sonar target detection. Conf Proc IEEE Eng Med Biol Soc 2010, 4444-4447
4. Backhaus, J., Hoeckesfeld, R., Born, J., Hohagen, F., and Junghanns, K. (2008) Immediate as well as delayed post learning sleep but not wakefulness enhances declarative memory consolidation in children. Neurobiol Learn Mem 89, 76-80
5. Badia, P., Myers, B., Boecker, M., Culpepper, J., and Harsh, J.R. (1991) Bright light effects on body temperature, alertness, EEG and behavior. Physiol Behav 50, 583-588
6. Bakker, A., Kirwan, C.B., Miller, M., and Stark, C.E. (2008) Pattern separation in the human hippocampal CA3 and dentate gyrus. Science 319, 1640-1642
7. Banks, S., and Dinges, D.F. (2007) Behavioral and physiological consequences of sleep restriction. J Clin Sleep Med 3, 519-528
8. Beebe, D.W., Difrancesco, M.W., Tlustos, S.J., McNally, K.A., and Holland, S.K. (2009) Preliminary fMRI findings in experimentally sleep-restricted adolescents engaged in a working memory task. Behav Brain Funct 5, 9
9. Benes, F.M., Turtle, M., Khan, Y., and Farol, P. (1994) Myelination of a key relay zone in the hippocampal formation occurs in the human brain during childhood, adolescence, and adulthood. Arch Gen Psychiatry 51, 477-484.
10. Biederman, J., Boellner, S.W., Childress, A., Lopez, F.A., Krishnan, S., and Zhang, Y. (2007) Lisdexamfetamine dimesylate and mixed amphetamine salts extended-release in children with ADHD: a double-blind, placebo-controlled, crossover analog classroom study. Biol Psychiatry 62, 970-976
11. Born, J., Rasch, B., and Gais, S. (2006) Sleep to remember. Neuroscientist 12, 410-424
12. Born, J., and Wagner, U. (2009) Sleep, hormones, and memory. Obstet Gynecol Clin North Am 36, 809-829, x
13. Carskadon, M.A. (1990) Patterns of sleep and sleepiness in adolescents. Pediatrician 17, 5-12
14. Carskadon, M.A., Acebo, C., and Jenni, O.G. (2004) Regulation of adolescent sleep: implications for behavior. Ann N Y Acad Sci 1021, 276-291
15. Carskadon, M.A., Harvey, K., and Dement, W.C. (1981) Sleep loss in young adolescents. Sleep 4, 299-312
16. Casey, B.J., Getz, S., and Galvan, A. (2008) The adolescent brain. Developmental Review 28, 62-77
17. Castellanos, F.X., Margulies, D.S., Kelly, C., Uddin, L.Q., Ghaffari, M., Kirsch, A., Shaw, D., Shehzad, Z., Di Martino, A., Biswal, B., Sonuga-Barke, E.J., Rotrosen, J., Adler, L.A., and Milham, M.P. (2008) Cingulate-precuneus interactions: a new locus of dysfunction in adult attention-deficit/hyperactivity disorder. Biol Psychiatry 63, 332-337
18. Chee, M.W., and Choo, W.C. (2004) Functional imaging of working memory after 24 hr of total sleep deprivation. J Neurosci 24, 4560-4567
19. Chee, M.W., and Chuah, L.Y. (2008) Functional neuroimaging insights into how sleep and sleep deprivation affect memory and cognition. Curr Opin Neurol 21, 417-423
20. Chee, M.W., and Chuah, Y.M. (2007) Functional neuroimaging and behavioral correlates of capacity decline in visual short-term memory after sleep deprivation. Proc Natl Acad Sci U S A 104, 9487-9492

21. Crowley, S.J., Acebo, C., and Carskadon, M.A. (2007) Sleep, circadian rhythms, and delayed phase in adolescence. Sleep Med 8, 602-612
22. Curcio, G., Ferrara, M., and De Gennaro, L. (2006) Sleep loss, learning capacity and academic performance. Sleep Med Rev 10, 323-337
23. Dewald, J.F., Meijer, A.M., Oort, F.J., Kerkhof, G.A., and Bogels, S.M. (2010) The influence of sleep quality, sleep duration and sleepiness on school performance in children and adolescents: A meta-analytic review. Sleep Med Rev 14, 179-189
24. Diekelmann, S., and Born, J. (2010) The memory function of sleep. Nat Rev Neurosci 11, 114-126
25. Dockree, P.M., Kelly, S.P., Foxe, J.J., Reilly, R.B., and Robertson, I.H. (2007) Optimal sustained attention is linked to the spectral content of background EEG activity: greater ongoing tonic alpha (approximately 10 Hz) power supports successful phasic goal activation. Eur J Neurosci 25, 900-907
26. Drummond, S.P., Brown, G.G., Gillin, J.C., Stricker, J.L., Wong, E.C., and Buxton, R.B. (2000) Altered brain response to verbal learning following sleep deprivation. Nature 403, 655-657
27. Drummond, S.P., Brown, G.G., Stricker, J.L., Buxton, R.B., Wong, E.C., and Gillin, J.C. (1999) Sleep deprivation-induced reduction in cortical functional response to serial subtraction. Neuroreport 10, 3745-3748
28. Durmer, J.S., and Dinges, D.F. (2005) Neurocognitive consequences of sleep deprivation. Semin Neurol 25, 117-129
29. Dworak, M., Schierl, T., Bruns, T., and Struder, H.K. (2007) Impact of singular excessive computer game and television exposure on sleep patterns and memory performance of school-aged children. Pediatrics 120, 978-985
30. Feinberg, I. (1982-3) Schizophrenia: Caused by a fault in programmed synaptic elimination during adolescence? Journal of Psychiatric Research 17, 319-334
31. Gais, S., Hullemann, P., Hallschmid, M., and Born, J. (2006) Sleep-dependent surges in growth hormone do not contribute to sleep-dependent memory consolidation. Psychoneuroendocrinology 31, 786-791
32. Gibson, E.S., Powles, A.C., Thabane, L., O'Brien, S., Molnar, D.S., Trajanovic, N., Ogilvie, R., Shapiro, C., Yan, M., and Chilcott-Tanser, L. (2006) "Sleepiness" is serious in adolescence: two surveys of 3235 Canadian students. BMC Public Health 6, 116
33. Giedd, J.N. (2008) The teen brain: Insights from neuroimaging. Journal of Adolescent Health 42, 335-343
34. Goel, N., Rao, H., Durmer, J.S., and Dinges, D.F. (2009) Neurocognitive consequences of sleep deprivation. Semin Neurol 29, 320-339
35. Greicius, M.D., Flores, B.H., Menon, V., Glover, G.H., Solvason, H.B., Kenna, H., Reiss, A.L., and Schatzberg, A.F. (2007) Resting-state functional connectivity in major depression: abnormally increased contributions from subgenual cingulate cortex and thalamus. Biol Psychiatry 62, 429-437
36. Gujar, N., Yoo, S.S., Hu, P., and Walker, M.P. (2010) The unrested resting brain: sleep deprivation alters activity within the default-mode network. J Cogn Neurosci 22, 1637-1648
37. Hochberg, Y., and Benjamini, Y. (1990) More powerful procedures for multiple significance testing. Stat Med 9, 811-818
38. Huttenlocher, P.R., and Dabholkar, A.S. (1997) Regional differences in synaptogenesis in human cerebral cortex. J Comp Neurol 387, 167-178
39. Jung, T.P., Makeig, S., Stensmo, M., and Sejnowski, T.J. (1997) Estimating alertness from the EEG power spectrum. IEEE Trans Biomed Eng 44, 60-69
40. Kirwan, C.B., and Stark, C.E. (2007) Overcoming interference: an fMRI investigation of pattern separation in the medial temporal lobe. Learn Mem 14, 625-633
41. Kopasz, M., Loessl, B., Hornyak, M., Riemann, D., Nissen, C., Piosczyk, H., and Voderholzer, U. (2010) Sleep and memory in healthy children and adolescents - a critical review. Sleep Med Rev 14, 167-177

42. Margulies, D.S., Kelly, A.M., Uddin, L.Q., Biswal, B.B., Castellanos, F.X., and Milham, M.P. (2007) Mapping the functional connectivity of anterior cingulate cortex. Neuroimage 37, 579-588
43. Matousek, M., and Petersen, I. (1983) A method for assessing alertness fluctuations from EEG spectra. Electroencephalogr Clin Neurophysiol 55, 108-113
44. O'Brien, L.M. (2009) The neurocognitive effects of sleep disruption in children and adolescents. Child Adolesc Psychiatr Clin N Am 18, 813-823
45. Ortega, F.B., Ruiz, J.R., Castillo, R., Chillon, P., Labayen, I., Martinez-Gomez, D., Redondo, C., Marcos, A., and Moreno, L.A. (2010) Sleep duration and cognitive performance in adolescence. The AVENA study. Acta Paediatr 99, 454-456
46. Pilcher, J.J., and Walters, A.S. (1997) How sleep deprivation affects psychological variables related to college students' cognitive performance. J Am Coll Health 46, 121-126
47. Randazzo, A.C., Muehlbach, M.J., Schweitzer, P.K., and Walsh, J.K. (1998) Cognitive function following acute sleep restriction in children ages 10-14. Sleep 21, 861-868
48. Roberts, R.E., Roberts, C.R., and Duong, H.T. (2009) Sleepless in adolescence: Prospective data on sleep deprivation, health and functioning. Journal of adolescence
49. Sadaghiani, S., Scheeringa, R., Lehongre, K., Morillon, B., Giraud, A.L., and Kleinschmidt, A. (2010) Intrinsic connectivity networks, alpha oscillations, and tonic alertness: a simultaneous electroencephalography/functional magnetic resonance imaging study. J Neurosci 30, 10243-10250
50. Sadeh, A., Alster, J., Urbach, D., and Lavie, P. (1989) Actigraphically based automatic bedtime sleep-wake scoring: validity and clinical applications. J Ambul Monitoring 2
51. Sadeh, A., Gruber, R., and Raviv, A. (2002) Sleep, neurobehavioral functioning, and behavior problems in school-age children. Child Dev 73, 405-417
52. Sadeh, A., Gruber, R., and Raviv, A. (2003) The effects of sleep restriction and extension on school-age children: what a difference an hour makes. Child Dev 74, 444-455
53. Sadeh, A., Raviv, A., and Gruber, R. (2000) Sleep patterns and sleep disruptions in school-age children. Dev Psychol 36, 291-301
54. Siddiqui, O., Hung, H.M., and O'Neill, R. (2009) MMRM vs. LOCF: a comprehensive comparison based on simulation study and 25 NDA datasets. J Biopharm Stat 19, 227-246
55. Steenari, M.R., Vuontela, V., Paavonen, E.J., Carlson, S., Fjallberg, M., and Aronen, E. (2003) Working memory and sleep in 6- to 13-year-old schoolchildren. J Am Acad Child Adolesc Psychiatry 42, 85-92
56. Suratt, P.M., Barth, J.T., Diamond, R., D'Andrea, L., Nikova, M., Perriello, V.A., Jr., Carskadon, M.A., and Rembold, C. (2007) Reduced time in bed and obstructive sleep-disordered breathing in children are associated with cognitive impairment. Pediatrics 119, 320-329
57. Teicher, M.H., Andersen, S.L., and Hostetter, J.C., Jr. (1995) Evidence for dopamine receptor pruning between adolescence and adulthood in striatum but not nucleus accumbens. Brain Res Dev Brain Res 89, 167-172
58. Teicher, M.H., Ito, Y., Glod, C.A., and Barber, N.I. (1996) Objective measurement of hyperactivity and attentional problems in ADHD. J Am Acad Child Adolesc Psychiatry 35, 334-342
59. Teicher, M.H., Lowen, S.B., Polcari, A., Foley, M., and McGreenery, C.E. (2004) Novel strategy for the analysis of CPT data provides new insight into the effects of methylphenidate on attentional states in children with ADHD. J Child Adolesc Psychopharmacol 14, 219-232
60. Teicher, M.H., Polcari, A., and McGreenery, C.E. (2008) Utility of objective measures of activity and attention in the assessment of therapeutic response to stimulants in children with attention-deficit/hyperactivity disorder. J Child Adolesc Psychopharmacol 18, 265-270
61. Thomas, M., Sing, H., Belenky, G., Holcomb, H., Mayberg, H., Dannals, R., Wagner, H., Thorne, D., Popp, K., Rowland, L., Welsh, A., Balwinski, S., and Redmond, D. (2000) Neural basis of alertness and cognitive performance impairments during sleepiness. I. Effects of 24 h of sleep deprivation on waking human regional brain activity. J Sleep Res 9, 335-352
62. Walker, M.P. (2008) Cognitive consequences of sleep and sleep loss. Sleep Med 9 Suppl 1, S29-34

63. Walker, M.P., and Stickgold, R. (2006) Sleep, memory, and plasticity. Annu Rev Psychol 57, 139-166
64. Yoo, S.S., Hu, P.T., Gujar, N., Jolesz, F.A., and Walker, M.P. (2007) A deficit in the ability to form new human memories without sleep. Nat Neurosci 10, 385-392
65. Williams, S.T. (2008) Review of Mental Health Screening and Assessment Tools. Northern California Training Academy,18
