# Supplementary material for: Bright light therapy and early morning attention, mathematical performance, electroencephalography and brain connectivity in adolescents with morning sleepiness
Source: PLoS One. 2023 Aug 22;18(8):e0273269. doi: 10.1371/journal.pone.0273269 (PMC10443881; doi:10.1371/journal.pone.0273269)
Supplement: S3 File — Results of the published statistical analyses from running the R scripts. (PDF) [file pone.0273269.s011.pdf]

````
---
title: "LiteBook Study"
author: "Martin H. Teicher MD PhD"
date: "4/25/2023"
output: html_document
---

```{r setup, include=FALSE}
knitr::opts_chunk$set(echo = TRUE)
```
## Introduction

Data and analyses for publication - Litebook Study.

# Load datasets
```{r echo=FALSE}
file1 <- "/Users/marty/Documents/R-working directory/S1_Litebook_Demo_PERMP_ESS.csv"
f1 <- read.csv(file1, header=T)
file2 <- "/Users/marty/Documents/R-working
directory/S2_LiteBook_SleepExportDetails_rev.csv"
f2 <- read.csv(file2, header=T)
file3 <- "/Users/marty/Documents/R-working
directory/S3_LiteBook_serial_addition_subtraction_task.csv"
f3 <- read.csv(file3, header=T)
file4 <- "/Users/marty/Documents/R-working
directory/S4_Litebook_Quotient_percent_change.csv"
f4 <- read.csv(file4, header=T)
file5 <- "/Users/marty/Documents/R-working directory/S5_Litebook_sleepiness_scale.csv"
f5 <- read.csv(file5, header=T)
file6 <- "/Users/marty/Documents/R-working
directory/S6_LiteBook_electrode_powerbands_EO_absolute.csv"
f6 <- read.csv(file6, header=T)
file7 <- "/Users/marty/Documents/R-working
directory/S7_LiteBook_electrode_powerbands_EC_absolute.csv"
f7 <- read.csv(file7, header=T)

```


# Load Libraries
```{r message=FALSE}
library(relaimpo)
library(nlme)
library(car)
library(lme4)
library(LMERConvenienceFunctions)
```
# Data
The original data file was: `r file2`
 + It consists of `r nrow(f1)` rows and `r ncol(f1)` columns
 + `r table(f1$Gender)`
 + Minimum age = `r min(f1$Age, na.rm=T)`
 + Maximum age = `r max(f1$Age, na.rm=T)`
 + Percent use mean = `r mean(f1$Per_use, na.rm=T)` ± `r sd(f1$Per_use, na.rm=T)`
 + Minimum use = `r min(f1$Per_use, na.rm=T)`
 + Maximum use = `r max(f1$Per_use, na.rm=T)`

# PERMP Performance
```{r echo=FALSE}
av1 <- lm(pchange_Post_PERMP ~ Per_use + Pre_PERMP, data=f1)
summary(av1)
Anova(av1, type=3)
calc.relimp(av1)
av1b <- lm(pchange_Post_PERMP ~ Per_use, data=f1)
anova(av1, av1b)
````
````

````
summary(av1b)
Anova(av1b, type=3)
```

# Serial Addition Subtraction Task
```{r echo=TRUE}
av2 <- lme(lat2 ~ per_use2 + scale(rslt2) + scale(I(rslt2^2)), random = ~ 1|subj2,
weights = varIdent(form = ~1 | cond2), method = "REML", data = f3)
summary(av2)
av2.ar1 <- update(av2, corr = corAR1(0.8, form = ~ 1 | subj2))
av2.arma <- update(av2,corr = corARMA(p = 1, q = 1, form = ~ 1 | subj2))
av2.car1 <- update(av2,corr = corCAR1(form = ~ 1 | subj2))
anova(av2, av2.ar1)
print("Significantly improved by ar1 correlation structure")
anova(av2, av2.arma)
print("Significantly improved by arma correlation structure")
anova(av2, av2.car1)
print("Significantly improved by car1 correlation structure")
print("Use arma correlation structure")
summary(av2.arma)
anova.lme(av2.arma, type = "marginal", adjustSigma = F)
av2b.arma <- lme(lat2 ~ per_use2 + scale(rslt2) + scale(I(rslt2^2)), random = ~ 1|subj2,
corr = corARMA(p = 1, q = 1, form = ~ 1 | subj2), method = "REML", data = f3)
anova(av2.arma, av2b.arma)
```
# Sleep - Bed Time, Rise Time, Total time in bed
```{r echo=TRUE}

f2 <- read.csv(file2, header=T)
f2_data <- subset(f2, valid==1)
f2_data <- subset(f2_data, subj !="LB019" & subj != "LB020")
dim(f2_data)

#  Adjust for times after midnight (e.g., 1 am) being later - not much earlier
#f2_data$hr_new.bedtime[f2_data$hr_new.bedtime<12]<- f2_data$hr_new.bedtime+24
q<-which(f2_data$hr_new.bedtime<12)
f2_data$hr_new.bedtime[q]<- f2_data$hr_new.bedtime[q]+24

print("Bedtimes")
av2 <- lme(hr_new.bedtime ~ use2 * weekend.holiday + Gender + Age + acro1_fix, random = ~
1| subj, weights = varIdent(form = ~ 1 | condition), method = "ML",data = f2_data)
print(summary(av2))
av2.ar1 <- update(av2, corr = corAR1(0.8, form = ~ 1 | subj))
#av2.arma <- update(av2,corr = corARMA(p = 1, q = 1, form = ~ 1 | subj))
av2.car1 <- update(av2,corr = corCAR1(form = ~ 1 | subj))
print(anova(av2, av2.ar1))
print("Not signifiantly improved by ar1 correlation structure")
print(anova(av2, av2.car1))
print("Not Signifiantly improved by cor1 correlation structure")
anova.lme(av2, type = "marginal", adjustSigma = F)
print("Can we eliminate gender and age? - yes")
av3 <- lme(hr_new.bedtime ~ use2 * weekend.holiday  + acro1_fix, random = ~ 1| subj,
weights = varIdent(form = ~1 | condition), method = "ML",data = f2_data)
summary(av3)
anova(av2, av3)
print("Can we eliminate use2 * weekend.holiday interaction? - yes")
av4 <- lme(hr_new.bedtime ~ use2 + weekend.holiday +  acro1_fix, random = ~ 1| subj,
weights = varIdent(form = ~1 | condition), method = "ML",data = f2_data)
summary(av4)
anova(av3, av4)
print("Fit REML")
anova.lme(av4, type = "marginal", adjustSigma = F)
av4.reml <- lme(hr_new.bedtime ~ use2 + weekend.holiday +  acro1_fix, random = ~ 1| subj,
weights = varIdent(form = ~1 | condition), method = "REML",data = f2_data)
summary(av4.reml)
````

```
anova.lme(av4.reml, type = "marginal", adjustSigma = F)
print("Are bed times more variable post light? - yes")
av4.unwt <- lme(hr_new.bedtime ~ use2 + weekend.holiday + acro1_fix, random = ~ 1| subj,
method = "ML",data = f2_data)
summary(av4.unwt)
anova(av4, av4.unwt)
print("Trend - bedtimes were less variable post light")

print("School days only")
f2_data.sch <- subset(f2_data, weekend.holiday==0)
f2_data.hol <- subset(f2_data, weekend.holiday==1)
av5 <- lme(hr_new.bedtime ~ use2 +  acro1_fix, random = ~ 1| subj, weights = varIdent(form
= ~1 | condition), method = "ML",data = f2_data.sch)
summary(av5)
av5.unwt <- lme(hr_new.bedtime ~ use2 +  acro1_fix, random = ~ 1| subj, method = "ML",data
= f2_data.sch)
summary(av5.unwt)
anova(av5, av5.unwt)
print("Yes - bedtimes were 0.6741993 less variable post light on school days, LR test
12.58555        4e-04*2")

print("Weeekends only")
av6 <- lme(hr_new.bedtime ~ use2 +  acro1_fix, random = ~ 1| subj, weights = varIdent(form
= ~1 | condition), method = "ML",data = f2_data.hol)
summary(av5)
av6.unwt <- lme(hr_new.bedtime ~ use2 +  acro1_fix, random = ~ 1| subj, method = "ML",data
= f2_data.hol)
summary(av6.unwt)
anova(av6, av6.unwt)
print("No - bedtimes were not less variable on weekends - holidays")

#####################################################################
print("Rise times")
f2 <- read.csv(file2, header=T)
f2_data <- subset(f2, valid==1)
f2_data <- subset(f2_data, subj !="LB019" & subj != "LB020")

q <- which(f2_data$hr_new.risetime > 20 | f2_data$hr_new.risetime< 4)
f2_data$hr_new.risetime[q] <- NA

av2 <- lme(hr_new.risetime ~ use2 * weekend.holiday + Gender + Age + acro1_fix, random = ~
1| subj, weights = varIdent(form = ~ 1 | condition), method = "ML",data = f2_data,
na.action=na.omit)
summary(av2)
av2.ar1 <- update(av2, corr = corAR1(0.8, form = ~ 1 | subj))
av2.arma <- update(av2,corr = corARMA(p = 1, q = 1, form = ~ 1 | subj))
av2.car1 <- update(av2,corr = corCAR1(form = ~ 1 | subj))
print(anova(av2, av2.ar1))
print("Not signifiantly improved by ar1 correlation structure")
print(anova(av2, av2.ar1))
print("Not signifiantly improved by arma correlation structure")
print(anova(av2, av2.car1))
print("Not Signifiantly improved by cor1 correlation structure")

print("Can we eliminate age? - yes")
av3 <- lme(hr_new.risetime ~ use2 * weekend.holiday + Gender + acro1_fix, random = ~ 1|
subj, weights = varIdent(form = ~ 1 | condition), method = "ML",data = f2_data,
na.action=na.omit)
summary(av3)
anova(av2, av3)
print("Can we eliminate interaction? - yes")
av4 <- lme(hr_new.risetime ~ use2 + weekend.holiday + Gender + acro1_fix, random = ~ 1|
subj, weights = varIdent(form = ~ 1 | condition), method = "ML",data = f2_data,
na.action=na.omit)
summary(av4)
```

```
anova(av4, av3)

av4.reml <- lme(hr_new.risetime ~ use2 + weekend.holiday + Gender + acro1_fix, random = ~
1| subj, weights = varIdent(form = ~ 1 | condition), method = "REML",data = f2_data,
na.action=na.omit)
anova.lme(av4.reml, type = "marginal", adjustSigma = F)

print("Are rie times less variable post light? - no")
av4.unwt <- lme(hr_new.risetime ~ use2 + weekend.holiday + Gender + acro1_fix, random = ~
1| subj, method = "ML",data = f2_data, na.action=na.omit)
summary(av4.unwt)
anova(av4, av4.unwt)

print("During school days only?")
f2_data.sch <- subset(f2_data, weekend.holiday==0)
f2_data.hol <- subset(f2_data, weekend.holiday==1)

av5 <- lme(hr_new.risetime ~ use2 + Gender + acro1_fix, random = ~ 1| subj, weights =
varIdent(form = ~ 1 | condition), method = "ML",data = f2_data.sch, na.action=na.omit)
summary(av5)
anova.lme(av5, type = "marginal", adjustSigma = F)
print("Are rie times less variable post light? - no")
av5.unwt <- lme(hr_new.risetime ~ use2 + Gender + acro1_fix, random = ~ 1| subj, method =
"ML",data = f2_data.sch, na.action=na.omit)
anova(av5, av5.unwt)

print("During weekends-hollidays only?")

av6 <- lme(hr_new.risetime ~ use2 + Gender + acro1_fix, random = ~ 1| subj, weights =
varIdent(form = ~ 1 | condition), method = "ML",data = f2_data.hol, na.action=na.omit)
summary(av6)
anova.lme(av6, type = "marginal", adjustSigma = F)
print("Are rie times less variable post light? - no")
av6.unwt <- lme(hr_new.risetime ~ use2 + Gender + acro1_fix, random = ~ 1| subj, method =
"ML",data = f2_data.hol, na.action=na.omit)
anova(av6, av6.unwt)


####################################################################
print("Total time in bed")
f2 <- read.csv(file2, header=T)
f2_data <- subset(f2, valid==1)
f2_data <- subset(f2_data, subj !="LB019" & subj != "LB020")
q <- which(f2_data$sleep_dur < 180)
f2_data$sleep_dur[q] <- NA

av2 <- lme(sleep_dur ~ use2 * weekend.holiday + Gender + Age + acro1_fix, random = ~ 1|
subj, weights = varIdent(form = ~ 1 | condition), method = "ML",data = f2_data,
na.action=na.omit)
summary(av2)
av2.ar1 <- update(av2, corr = corAR1(0.8, form = ~ 1 | subj))
av2.arma <- update(av2,corr = corARMA(p = 1, q = 1, form = ~ 1 | subj))
av2.car1 <- update(av2,corr = corCAR1(form = ~ 1 | subj))
print(anova(av2, av2.ar1))
print("Not signifiantly improved by ar1 correlation structure")
print(anova(av2, av2.ar1))
print("Not signifiantly improved by arma correlation structure")
print(anova(av2, av2.car1))
print("Not Signifiantly improved by cor1 correlation structure")

print("Can we eliminate age and acrophase? - yes")
av3 <- lme(sleep_dur~ use2 * weekend.holiday + Gender, random = ~ 1| subj, weights =
varIdent(form = ~ 1 | condition), method = "ML",data = f2_data, na.action=na.omit)
summary(av3)
anova(av2, av3)
```

````
print("Can we remove interaction?  yes")
av4 <- lme(sleep_dur~ use2 + weekend.holiday + Gender, random = ~ 1| subj, weights = varIdent(form = ~ 1 | condition), method = "ML",data = f2_data, na.action=na.omit)
summary(av4)
anova(av4, av3)

av4.reml <- lme(sleep_dur~ use2 + weekend.holiday + Gender, random = ~ 1| subj, weights = varIdent(form = ~ 1 | condition), method = "REML",data = f2_data, na.action=na.omit)
anova.lme(av4.reml, type="marginal", adjustSigma=FALSE)

print("Is time in bed less variable post Litebook?")
av4.unwt <- lme(sleep_dur~ use2 + weekend.holiday + Gender, random = ~ 1| subj, method = "ML",data = f2_data, na.action=na.omit)
summary(av4.unwt)
anova(av4, av4.unwt)
print("Trend for post lite sleep duration 0.8475573  to be less variable")

print("During school days only?")
f2_data.sch <- subset(f2_data, weekend.holiday==0)
f2_data.hol <- subset(f2_data, weekend.holiday==1)

av5 <- lme(sleep_dur~ use2 + Gender, random = ~ 1| subj, weights = varIdent(form = ~ 1 | condition), method = "ML",data = f2_data.sch, na.action=na.omit)
summary(av5)
av5.unwt <- lme(sleep_dur~ use2 + Gender, random = ~ 1| subj, method = "ML",data = f2_data.sch, na.action=na.omit)
summary(av5.unwt)
anova(av5, av5.unwt)
print("Post light sleep duration reduction 0.6421011 is significant LR = 14.37212 2e-04")

print("During weekend only?")
av6 <- lme(sleep_dur~ use2 + Gender, random = ~ 1| subj, weights = varIdent(form = ~ 1 | condition), method = "ML",data = f2_data.hol, na.action=na.omit)
summary(av6)
av6.unwt <- lme(sleep_dur~ use2 + Gender, random = ~ 1| subj,  method = "ML",data = f2_data.hol, na.action=na.omit)
summary(av6.unwt)
anova(av6,av6.unwt)
print("No evidence for more consistent sleep during weekens post light")


```


# Sleep - Onset
```{r echo=TRUE}

###### Using Cole-Kripke algorithm
print("Sleep onset Cole")

f2 <- read.csv(file2, header=T)
f2_data <- subset(f2, valid==1)
f2_data <- subset(f2_data, subj !="LB019" & subj != "LB020")
#  Adjust for times after midnight (e.g., 1 am) being later - not much earlier
#f2_data$hr_new.bedtime[f2_data$hr_new.bedtime<12]<- f2_data$hr_new.bedtime+24

q3<- which(f2_data$sleep_onset_Cole<12)
f2_data$sleep_onset_Cole[q3] <- f2_data$sleep_onset_Cole[q3]+24
q4 <- which(f2_data$sleep_onset_Cole > 40)
f2_data$sleep_onset_Cole[q4] <- f2_data$sleep_onset_Cole[q4]-24

av2 <- lme(sleep_onset_Cole ~ use2 * weekend.holiday + Gender + Age + acro1_fix, random = ~ 1| subj, weights = varIdent(form = ~ 1 | condition), method = "ML",data = f2_data)
print(summary(av2))
````

```
av2.ar1 <- update(av2, corr = corAR1(0.8, form = ~ 1 | subj))
av2.arma <- update(av2,corr = corARMA(p = 1, q = 1, form = ~ 1 | subj))
av2.car1 <- update(av2,corr = corCAR1(form = ~ 1 | subj))
print(anova(av2, av2.ar1))
print("Not signifiantly improved by ar1 correlation structure")
print(anova(av2, av2.arma))
print("Yes, signifiantly improved by arma correlation structure p = .048")
print(anova(av2, av2.car1))
print("Not signifiantly improved by cor1 correlation structure")

summary(av2.arma)

print("Can we eliminate age and gender? - yes")
av3 <- lme(sleep_onset_Cole ~ use2 * weekend.holiday + acro1_fix, random = ~ 1| subj,
weights = varIdent(form = ~1 | condition), corr = corARMA(p = 1, q = 1, form = ~ 1 |
subj), method = "ML",data = f2_data)
summary(av3)
anova(av3, av2.arma)

print("Can we remove interaction?- yes")
av4 <- lme(sleep_onset_Cole ~ use2 + weekend.holiday + acro1_fix, random = ~ 1| subj,
weights = varIdent(form = ~1 | condition), corr = corARMA(p = 1, q = 1, form = ~ 1 |
subj), method = "ML",data = f2_data)
summary(av4)
anova(av4, av3)

av4.reml <- lme(sleep_onset_Cole ~ use2 + weekend.holiday + acro1_fix, random = ~ 1| subj,
weights = varIdent(form = ~1 | condition), corr = corARMA(p = 1, q = 1, form = ~ 1 |
subj), method = "REML",data = f2_data)
anova.lme(av4.reml, type="marginal", adjustSigma=FALSE)

print("Is sleep onset more consistent post light - 0.8524633 ")
av5 <- lme(sleep_onset_Cole ~ use2 + weekend.holiday + acro1_fix, random = ~ 1| subj, corr
= corARMA(p = 1, q = 1, form = ~ 1 | subj), method = "ML",data = f2_data)
summary(av5)
anova(av5, av4)
print("Yes, sleep onset less variable post litebook -variance = 0.8407181, LR = 8.468232
LR = 0.0036")

print("On school days only?")
f2_data.sch <- subset(f2_data, weekend.holiday==0)
f2_data.hol <- subset(f2_data, weekend.holiday==1)

av6 <- lme(sleep_onset_Cole ~ use2  + acro1_fix, random = ~ 1| subj, weights =
varIdent(form = ~1 | condition), corr = corARMA(p = 1, q = 1, form = ~ 1 | subj), method =
"ML",data = f2_data.sch)
summary(av6)
av6.unwt <- lme(sleep_onset_Cole ~ use2 + acro1_fix, random = ~ 1| subj, corr = corARMA(p
= 1, q = 1, form = ~ 1 | subj), method = "ML",data = f2_data.sch)
summary(av6.unwt)
anova(av6, av6.unwt)
print("Yes - significantly less variable 0.6921826 post light, LR - 11.86724    6e-04")

print("On weekends or holidays?")

av7 <- lme(sleep_onset_Cole ~ use2  + acro1_fix, random = ~ 1| subj, weights =
varIdent(form = ~1 | condition), corr = corARMA(p = 1, q = 1, form = ~ 1 | subj), method =
"ML",data = f2_data.hol)
summary(av6)
av7.unwt <- lme(sleep_onset_Cole ~ use2 + acro1_fix, random = ~ 1| subj,corr = corARMA(p =
1, q = 1, form = ~ 1 | subj), method = "ML",data = f2_data.hol)
summary(av7.unwt)
anova(av7, av7.unwt)
print("No, not- significantly less variable 0.6921826  - post light")
```

````
# Sleep - Total Sleep Time
```{r echo=TRUE}

q3 <- which(f2_data$total_sleep_Cole < 200)
f2_data$total_sleep_Cole[q3] <- NA


av2 <- lme(total_sleep_Cole ~ use2 * weekend.holiday + Age + Gender, random = ~ 1| subj,
weights = varIdent(form = ~1 | condition), method = "ML",data = f2_data,
na.action=na.omit)
summary(av2)
av2.ar1 <- update(av2, corr = corAR1(0.8, form = ~ 1 | subj))
av2.arma <- update(av2,corr = corARMA(p = 1, q = 1, form = ~ 1 | subj))
av2.car1 <- update(av2,corr = corCAR1(form = ~ 1 | subj))
anova(av2, av2.ar1)
print("Not significantly improved by ar1 correlation structure")
anova(av2, av2.arma)
print("Not significantly improved by arma correlation structure")
anova(av2, av2.car1)
print("Not significantly improved by car1 correlation structure")

# Can we eliminate age? - Yes
av3 <- lme(total_sleep_Cole ~ use2 * weekend.holiday + Gender, random = ~ 1| subj, weights
= varIdent(form = ~1 | condition), method = "ML",data = f2_data, na.action=na.omit)
summary(av3)
anova(av2, av3)

# Can we eliminate interaction effects? - Yes
av4 <- lme(total_sleep_Cole ~ use2 + weekend.holiday + Gender, random = ~ 1| subj, weights
= varIdent(form = ~1 | condition), method = "ML",data = f2_data, na.action=na.omit)
summary(av4)
anova(av3, av4)
# Calculate using REML
av4.reml <- lme(total_sleep_Cole ~ use2 + weekend.holiday + Gender, random = ~ 1| subj,
weights = varIdent(form = ~1 | condition), method = "REML",data = f2_data,
na.action=na.omit)
summary(av4.reml)
anova.lme(av4.reml, type = "marginal", adjustSigma = F)

print("Was total sleep time more consistent with BLT?")
av4.unwt <- lme(total_sleep_Cole ~ use2 + weekend.holiday + Gender, random = ~ 1|
subj,method = "ML",data = f2_data, na.action=na.omit)
anova(av4, av4.unwt)
print("No, total sleep variability 0.8467083, trend level difference - LR =3.269501, p =
0.0706")

print("School days only")
f2_data.sch <- subset(f2_data, weekend.holiday==0)
f2_data.hol <- subset(f2_data, weekend.holiday==1)

av5 <- lme(total_sleep_Cole ~ use2 + Gender, random = ~ 1| subj, weights = varIdent(form =
~1 | condition), method = "ML",data = f2_data.sch, na.action=na.omit)
summary(av5)
print("Was total sleep time more consistent with BLT?")
av5.unwt <- lme(total_sleep_Cole ~ use2 + Gender, random = ~ 1| subj, method = "ML",data =
f2_data.sch, na.action=na.omit)
summary(av5.unwt)
anova(av5, av5.unwt)
print("Yes, reduction in variablity 0.6460155 was significant - LR = 13.80601   p = 2e-
04")

print("Weekends-holidays  only")
````
````

````
av6 <- lme(total_sleep_Cole ~ use2 + Gender, random = ~ 1| subj, weights = varIdent(form =
~1 | condition), method = "ML",data = f2_data.hol, na.action=na.omit)
summary(av5)
print("Was total sleep time more consistent with BLT?")
av6.unwt <- lme(total_sleep_Cole ~ use2 + Gender, random = ~ 1| subj, method = "ML",data =
f2_data.hol, na.action=na.omit)
summary(av6.unwt)
anova(av6, av6.unwt)
print("No, reduction in variablity 0.6460155 was not significant")

```
# Sleep Efficienty
```{r echo=TRUE}
print("sleep_eff_Cole")
q3 <- which(f2_data$sleep_eff_Cole < 0.8)
f2_data$sleep_eff_Cole[q3] <- NA

av2 <- lme(sleep_eff_Cole ~ use2 * weekend.holiday + Age + Gender + acro1_fix, random = ~
1| subj, weights = varIdent(form = ~1 | condition), method = "ML",na.action=na.omit, data
= f2_data)
summary(av2)
av2.ar1 <- update(av2, corr = corAR1(0.8, form = ~ 1 | subj))
av2.arma <- update(av2,corr = corARMA(p = 1, q = 1, form = ~ 1 | subj))
av2.car1 <- update(av2,corr = corCAR1(form = ~ 1 | subj))
anova(av2, av2.ar1)
print("Not improved by ar1 correlation structure")
anova(av2, av2.arma)
print("Not improved by arma correlation structure")
anova(av2, av2.car1)
print("Not improved by car1 correlation structure")

print("Can we eliminate age and gender? - Yes")
av3 <- lme(sleep_eff_Cole ~ use2 * weekend.holiday + acro1_fix, random = ~ 1| subj,
weights = varIdent(form = ~1 | condition), method = "ML",na.action=na.omit, data =
f2_data)
summary(av3)
anova(av2, av3)

print("Can we eliminate interaction? - yes")
av4 <- lme(sleep_eff_Cole ~ use2 + weekend.holiday + acro1_fix, random = ~ 1| subj,
weights = varIdent(form = ~1 | condition), method = "ML",na.action=na.omit, data =
f2_data)
summary(av4)
anova(av3, av4)

av4.reml <- lme(sleep_eff_Cole ~ use2 + weekend.holiday + acro1_fix, random = ~ 1| subj,
weights = varIdent(form = ~1 | condition), method = "REML",na.action=na.omit, data =
f2_data)
anova.lme(av4.reml, type="marginal", adjustSigma=FALSE)

print("Is sleep efficiency more consistent with BLT?")
av4.unwt <- lme(sleep_eff_Cole ~ use2 + weekend.holiday + acro1_fix, random = ~ 1| subj,
method = "ML",na.action=na.omit, data = f2_data)
summary(av4.unwt)
anova(av4, av4.unwt)
print("No - not significantly less variable")

print("School days only")
f2_data.sch <- subset(f2_data, weekend.holiday==0)
f2_data.hol <- subset(f2_data, weekend.holiday==1)

av5 <- lme(sleep_eff_Cole ~ use2 + acro1_fix, random = ~ 1| subj, weights = varIdent(form
= ~1 | condition), method = "ML",na.action=na.omit, data = f2_data.sch)
summary(av5)
av5.unwt <- lme(sleep_eff_Cole ~ use2 + acro1_fix, random = ~ 1| subj, method =
````

````
"ML",na.action=na.omit, data = f2_data.sch)
summary(av5.unwt)
anova(av5, av5.unwt)
```
# Epworth Sleepiness Scale
```{r echo=TRUE}
av1 <- lme(ESS ~ Per_use + Gender + Age, random = ~ 1|Subject.Number, weights =
varIdent(form = ~1 | Condition), method = "ML", na.action=na.omit, data = f5)
summary(av1)
# Can we eliminate age? - Yes
av2 <- lme(ESS ~ Per_use + Gender, random = ~ 1|Subject.Number, weights = varIdent(form =
~1 | Condition), method = "ML", na.action=na.omit, data = f5)
summary(av2)
anova(av1, av2)
# Can we eliminate geder? - Yes
av3 <- lme(ESS ~ Per_use, random = ~ 1|Subject.Number, weights = varIdent(form = ~1 |
Condition), method = "ML", na.action=na.omit, data = f5)
summary(av3)
anova(av2, av3)
# Use REML
av3b <- lme(ESS ~ Per_use, random = ~ 1|Subject.Number, weights = varIdent(form = ~1 |
Condition), method = "REML", na.action=na.omit, data = f5)
summary(av3b)
anova.lme(av3b, type = "marginal", adjustSigma = F)
# Is weighting helpful? - No
av3c <- lme(ESS ~ Per_use, random = ~ 1|Subject.Number, method = "ML", na.action=na.omit,
data = f5)
summary(av3c)
anova(av3, av3c)
# Use REML
av3d <- lme(ESS ~ Per_use, random = ~ 1|Subject.Number, method = "REML",
na.action=na.omit, data = f5)
summary(av3d)
anova.lme(av3d, type = "marginal", adjustSigma = F)

names(f5)

f5.1 <- subset(f5, Condition=="PRE")
f5.2 <- subset(f5, Condition=="POST")
q <- which(f5.1$ESS >= 11)
f5.1$ESS[q]
f5.2$ESS[q]
q <- c(4,5,7,14,15,16,18,19,21,23,25)
t.test(as.numeric(f5.1$ESS[q]),y=as.numeric(f5.2$ESS[q]))
library(lsr)
cohensD(as.numeric(f5.1$ESS[q]),y=as.numeric(f5.2$ESS[q]))

hist(f5.2$Per_use, main = "Histogram of Percent Use",ylab="Number of
participants",xlab="Precent Use")

mean(f5.1$ESS, na.rm=TRUE)

```

# Quotient Accuracy
```{r echo=FALSE}
av1 <- lm(scale(pchnge_Acc) ~ scale(Per_use) + scale(Acc), f4)
summary(av1)
calc.relimp(av1)
```
# Quotient Errors of Omission
```{r echo=FALSE}
av1 <- lm(scale(pchnge_OmErr) ~ scale(Per_use) + scale(OmErr), f4)
summary(av1)
calc.relimp(av1)
````

~~~~
```
# Quotient Errors of Commission
```{r echo=FALSE}
av1 <- lm(scale(pchnge_ComErr) ~ scale(Per_use) + scale(ComErr), f4)
summary(av1)
calc.relimp(av1)
```
# Quotient Latency
```{r echo=FALSE}
av1 <- lm(scale(pchnge_Latency) ~ scale(Per_use) + scale(Latency), f4)
summary(av1)
calc.relimp(av1)
```
# Quotient Variability
```{r echo=FALSE}
av1 <- lm(scale(pchnge_Var) ~ scale(Per_use) + scale(Var), f4)
summary(av1)
calc.relimp(av1)
```
# Quotiet Immobility
```{r echo=FALSE}
av1 <- lm(scale(pchnge_ImmDur_perc) ~ scale(Per_use) + scale(ImmDur_perc), f4)
summary(av1)
calc.relimp(av1)
```


## EEG Analyses
# subroutine
```{r echo=TRUE}
EEG_calc_lmer_percents<- function(f6, c1, cn, per_use = c(50,80)){
        # f6 - EEG power spectrum file
        # c1 - start column for EEG file
        # c2 - end column
        # per_use - to estimate what EEG spectra would be at different levels of use
        lpu <- length(per_use)
        rslt <- rep(NA,(cn-c1+1)*(lpu+5))
        dim(rslt) <- c(columns=(lpu+5), rows=(cn-c1+1))
        cnames <- c("Measure","Baseline")
                for(i in c1:cn) {
                indx <- i - c1 + 1
                hold <- f6[,i]
                lmm.2 <- lmer(formula = hold ~ Per_Use2 + (1|SUBJ), data = f6, REML =
TRUE, verbose = FALSE)
                zzz <- summary(lmm.2)
                zz2 <- pamer.fnc(lmm.2, ndigits = 4)
                rslt[1,indx] <- zzz$coefficients[1,1]
                for(j in 1:lpu) {
                        rslt[(j+1),indx] <- zzz$coefficients[1,1] + zzz$coefficients[2,1]
* per_use[j]
                        if(i==c1) {cnames <- c(cnames,paste(per_use[j],"% use",sep=""))}
                }
                #rslt[(lpu+2),indx] <- zzz$coefficients[2,3]
                rslt[(lpu+2),indx] <- zz2$`F value`
                rslt[(lpu+3),indx] <- zz2$lower.den.df
                rslt[(lpu+4),indx] <- zz2$lower.p.val
                rslt[(lpu+5),indx] <- zz2$expl.dev
        }
        cnames <- c(cnames,"F value","lower df","P-val","Per_var")
        out <- data.frame(names(f6)[c1:cn], t(rslt))
        names(out) <- cnames
        return(out)
}
```
# Eyes open - Delta
```{r echo=FALSE}
~~~~

~~~~
xxx <- EEG_calc_lmer_percents(f6,c1=5,cn=23,per_use=c(50,80))
print(xxx)
```
# Eyes open - Theta
```{r echo=FALSE}
xxx <- EEG_calc_lmer_percents(f6,c1=24,cn=42,per_use=c(50,80))
print(xxx)
```
# Eyes open - Alpha
```{r echo=FALSE}
xxx <- EEG_calc_lmer_percents(f6,c1=43,cn=61,per_use=c(50,80))
print(xxx)
```
# Eyes open - Beta1
```{r echo=FALSE}
xxx <- EEG_calc_lmer_percents(f6,c1=62,cn=80,per_use=c(50,80))
print(xxx)
```
# Eyes open - Beta2
```{r echo=FALSE}
xxx <- EEG_calc_lmer_percents(f6,c1=81,cn=99,per_use=c(50,80))
print(xxx)
```
# Eyes open - Gamma
```{r echo=FALSE}
xxx <- EEG_calc_lmer_percents(f6,c1=100,cn=118,per_use=c(50,80))
print(xxx)
```
# Eyes closed - Delta
```{r echo=FALSE}
xxx <- EEG_calc_lmer_percents(f7,c1=5,cn=23,per_use=c(50,80))
print(xxx)
```
# Eyes closed - Theta
```{r echo=FALSE}
xxx <- EEG_calc_lmer_percents(f7,c1=24,cn=42,per_use=c(50,80))
print(xxx)
```
# Eyes closed - Alpha
```{r echo=FALSE}
xxx <- EEG_calc_lmer_percents(f7,c1=43,cn=61,per_use=c(50,80))
print(xxx)
```
# Eyes closed - Beta1
```{r echo=FALSE}
xxx <- EEG_calc_lmer_percents(f7,c1=62,cn=80,per_use=c(50,80))
print(xxx)
```
# Eyes closed - Beta2
```{r echo=FALSE}
xxx <- EEG_calc_lmer_percents(f7,c1=81,cn=99,per_use=c(50,80))
print(xxx)
```
# Eyes closed - Gamma
```{r echo=FALSE}
xxx <- EEG_calc_lmer_percents(f7,c1=100,cn=118,per_use=c(50,80))
print(xxx)
```
~~~~
